# “Bridging Guidelines and Real World Barriers: Reflections on GDMT Optimization in Heart Failure”

**DOI:** 10.1002/clc.70206

**Published:** 2025-09-10

**Authors:** Ibadullah Tahir, Hunain Shahbaz, Aimal Khattak

**Affiliations:** ^1^ Ayub Medical College Abbottabad Abbottabad Pakistan

Dear Editor,

Velasco et al. address a critical issue in contemporary HF care by examining titration limiting adverse effects (AEs) in a specialized guideline directed medical therapy (GDMT) optimization program [1]. In their single center cohort (*n* = 254 completers), 59% of patients encountered ≥ 1 AE (hypotension, bradycardia, hyperkalemia, renal dysfunction) hindering GDMT titration [1]. Notably, younger, nonischemic patients more often reached target doses. These observations underscore the reality that, despite guidelines advocating quadruple therapy (ARNi/ACEi/ARB, beta‐blockers, MRA, SGLT2i) for HFrEF [3] real world implementation lags: fewer than one in five eligible patients currently receive all four classes [5].

Velasco et al. are to be commended for quantifying AE rates within an intensive titration program and for analyzing predictors of submaximal dosing (e.g., older age and atrial fibrillation predicted lower beta blocker titration) [1]. This granular approach highlights important heterogeneity: for example, patients without hypertension or with atrial fibrillation had more difficulty up titrating renin angiotensin blockers [1]. Such findings point to the need for personalized strategies. However, the study's retrospective, single center design and focus on program completers may limit generalizability. Patients who discontinued the program (perhaps due to severe intolerance) were excluded; thus the true burden of AEs may be underestimated. Only four AE domains were assessed, omitting others (e.g., volume depletion, gastrointestinal symptoms, or device‐related issues) that can also limit GDMT. Finally, standardized definitions of “titration limiting” AEs were not detailed. As the authors note, establishing uniform AE criteria is essential for comparing programs and guiding practice [1].

Importantly, Velasco's results should be interpreted alongside broader barriers to GDMT uptake. Systematic reviews and registry data have documented multifactorial obstacles including clinician inertia, patient comorbidities, socioeconomic factors, and health system challenges that suppress GDMT use [2, 5]. The 2022 AHA/ACC/HFSA guideline reiterates that quadruple GDMT markedly improves survival and symptoms, yet emphasizes tailoring therapy to individual patient risk/benefit profiles [3]. Similarly, recent ACC consensus guidance highlights “special cohorts” (older adults, frailty, coexisting organ dysfunction, and socioeconomic barriers) that necessitate flexible GDMT strategies [6]. In this light, Velasco's finding that obesity was associated with higher titration success may reflect survivor bias or phenotype differences, and warrants further study.

Where Velasco et al. add value is by quantifying how often AEs intrude on a dedicated titration effort. Their high AE rate parallels other quality improvement experiences: for example, a nurse/pharmacist led titration program achieved target GDMT in 80% of patients by 6 months [6] but only after intensive follow‐up and patient education. Likewise, a recent pilot trial of a remote monitoring titration clinic showed significant improvements in GDMT “score” versus usual care [4] suggesting that innovative care models can partially overcome barriers. Integrating such multidisciplinary or telehealth approaches could mitigate some AEs by allowing more frequent monitoring and dose adjustments.

## Future Priorities

Should include prospective studies that (1) incorporate patient reported outcomes and quality of life metrics, (2) evaluate strategies to safely accelerate GDMT (as exemplified by the STRONG HF trial's outcomes), and (3) develop decision support tools to flag at‐risk patients. Velasco et al. rightly call for consensus on defining GDMT related AEs [1]; in parallel, we need predictive models that identify who will benefit from slower versus faster titration. Importantly, addressing non AE barriers (medication access, adherence support, care coordination) will be equally critical. In summary, Velasco et al. provide valuable real world data on the complexity of GDMT optimization [1]. Their findings remind us that human factors from physiology to behavior shape GDMT implementation. By acknowledging these nuances while continuing to pursue intensive titration, the HF community can advance toward truly patient centered care.

## Author Contributions

All authors have read and approved the final version of manuscript.

## Ethics Statement

The authors have nothing to report.

## Consent

The authors have nothing to report.

## Conflicts of Interest

The authors declare no conflicts of interest.

## Data Availability

The authors have nothing to report.
